# Additional effect of high-intensity laser therapy with conventional physiotherapy related to pain and function in patients with knee osteoarthritis: a randomized, double-blind, placebo-controlled, 12-week follow-up study

**DOI:** 10.1007/s10103-025-04516-6

**Published:** 2025-06-07

**Authors:** Selen Özgözen, Alaaddin Levent Özgözen, Pınar Doruk Analan, Caner İncekaş

**Affiliations:** 1 Department of Physical Medicine and Rehabilitation, Başkent University Faculty of Medicine, Dr. Turgut Noyan Training and Research Center, Adana, Turkey; 2 Department of Orthopedics and Traumatology, Başkent University Faculty of Medicine, Dr. Turgut Noyan Training and Research Center, Adana, Turkey; 3 Department of Biostatistics, Başkent University Faculty of Medicine, Ankara, Turkey

**Keywords:** Knee osteoarthritis, High-intensity laser therapy, Pain, Physiotherapy

## Abstract

**Objective:**

High-intensity laser therapy (HILT) is a relatively new form of Nd: YAG laser. The aim of the study is to investigate the additional benefits of HILT with conventional physiotherapy, related to pain and function, in patients with knee osteoarthritis (KOA).

**Method:**

The study comprised 43 knees from 31 patients of both genders with mean age 54.6 ± 6.22 (41–64) years. 53.49% of the knees were Kellgren Lawrence (KL) grade 2, and rest were KL grade 3 KOA. Group 1 (*n* = 21) received transcutaneous electrical nerve stimulation (TENS), hot packs (HP), exercises (EX), and HILT (Nd: yag-laser, 10 W). Group 2 (*n* = 22), received the same interventions but placebo HILT. All interventions were applied for 10 sessions. The Visual Analog Scale (VAS), Western Ontario & McMaster Universities Osteoarthritis Questionnaire (WOMAC), and Lequesne Algofunctional Index (LAI) were administered before, after, and at 12-week follow-up.

**Results:**

Baseline VAS, WOMAC, and LAI scores of the groups were similar (*p* > 0.05). After treatment and 12 weeks of follow-up, both groups had significant relief for VAS, WOMAC, LAI pain (respectively, *p* < 0.001) and function (*p* < 0.012), except LAI-walking distance (*p* = 0.415). Post-hoc analyses and mixed-effects models showed no significant differences between groups over time for all variables.

**Conclusions:**

HILT did not provide additional short- or mid-term benefits in pain or function when added to a conventional physiotherapy and exercise program in patients with stage 2 or 3 knee osteoarthritis under 65 years of age.

## Key points

• Though high-intensity laser therapy (HILT) is promising among current physiotherapy options for knee osteoarthritis (KOA), there is currently no consensus about treatment efficacy, optimal application dose and frequency of sessions.

• The inclusion of HILT to conventional physiotherapy and daily exercises for individuals with stage 2 or 3 KOA aged between 40-65 years, does not appear to provide any additional positive contribution to pain, stiffness and functionality.

## Introduction

Knee osteoarthritis (KOA) is a global cause of disability and chronic pain [1], and patients often attend physical therapy and orthopedic clinics. Core treatments for KOA include exercises, weight loss and maintenance, self-efficacy and self-management programs, and exercise. Various exercise therapies, such as walking, strengthening, neuromuscular training, aquatic exercises, yoga, and Tai Chi, may be recommended to patients without superiority over each other [2]. In daily practice, physical therapy modalities such as superficial thermotherapies (cold or hot packs), analgesic therapies (transcutaneous electrical nerve stimulation (TENS), interferential current), deep thermotherapies (such as ultrasound therapy and microwave therapy), photobiomodulation via laser therapy modalities, and neuromuscular electrical stimulation (NMES) are frequently used to regulate pain and functional recovery for KOA. Despite the frequent use of these physical therapy modalities for knee pain and functional improvements, there are several trials ongoing in order to reach consensus on treatment frequencies, doses, and clinical efficacies [3, 4].

Neodymium-doped yttrium aluminum garnet (Nd: YAG) is a laser type that emits light in the infrared region with a wavelength of 1064 nm. Low-level laser therapy (LLLT), using wavelengths between 640 and 905 nm, is widely used for treating musculoskeletal disorders, including knee osteoarthritis (KOA), due to its analgesic, anti-inflammatory, and photobiomodulatory effects [5–7]. Several studies and meta-analyses have reported that LLLT, either alone or in combination with exercise therapy, may lead to improvements in pain, muscle strength, and functional capacity in KOA patients [8, 9]. High-intensity laser therapy (HILT) is a newer Nd: YAG-based modality that delivers higher power outputs, potentially enhancing biological responses in deeper tissues. HILT has gained attention for its promising photochemical, biostimulatory, analgesic, and rapid anti-inflammatory properties [10–14]. While HILT is sometimes claimed to penetrate tissues more deeply than LLLT [15, 16], recent ex vivo comparisons suggest that the difference in penetration depth between 904/905 nm and 1064 nm lasers is relatively small and may not be clinically meaningful [17]. In the context of KOA, recent studies have shown increased clinical use of HILT. Some systematic reviews and meta-analyses have highlighted the potential benefits of HILT in improving pain, stiffness, joint mobility, muscle strength, functional capacity, and even cartilage structure [10, 13, 14]. However, it is worth noting that the majority of available HILT studies have short-term follow-up (≤ 6 weeks) [15, 18–22] and limited double-blind design [16, 21, 23], necessitating further robust investigations.

The research focused on determining whether HILT offers an additional advantage to the combined use of hot packs, TENS, and exercise in promoting short- and mid-term pain relief and functional recovery among patients with KOA.

## Materials and methods

This prospective, randomized, double-blind, and placebo-controlled study was performed in the Physical Medicine Rehabilitation (PMR) unit of Başkent University Dr. Turgut Noyan Educational Research Center in Adana between May 2022 and November 2023. The study was performed in accordance with the ethical standards for human research established by the Declaration of Helsinki and Good Clinical Practice guidelines and was approved by Başkent University Institutional Review Board and Ethics Committee (project no: KA22/129, date: 25 May 2022). In reporting the study protocol, the Consolidated Standards of Reporting Trials (CONSORT-2010) recommendations were taken into account. The study was retrospectively registered with clinicaltrials.gov identifier NCT06549543.

Patients with knee pain visiting the orthopedic or PMR specialist authors of this study were pre-evaluated, and those eligible and volunteer provided written informed consent. They were included if they (1) were both sexes and aged between 40 and 65 years; (2) had pain for at least 3 months in single or both knees; (3) were diagnosed as having KOA with Kellgren Lawrence (KL) Grade 2 or 3 KOA on weight-bearing anteroposterior X-ray images; (4) had normal serum acute phase reactants and uric acid levels; and (5) had no major effusion in the joint. They were excluded if they had a history of (1) therapeutic joint injection in the last 6 months, (2) physical therapy and/or HILT in the last 3 months, (3) any surgical invention for the knee joint, and (4) malignancy in the last 5 years.

### Sample size

The minimum sample size required for the study was calculated as a total of 36 knees, 18 knees in each group, with an effect size of 0.25 for the “ANOVA with repeated measures” method, with 90% test power, and 95% confidence level [21].

### Randomization

Participants were allocated to either the active HILT or placebo HILT group in a 1:1 ratio, following a pre-determined assignment sequence recorded by an independent researcher who was not involved in recruitment or assessment procedures. Although the allocation followed an alternating structure (i.e., every other participant assigned to each group), the full sequence was documented in an opaque notebook that was kept inaccessible to the investigators. The researchers responsible for recruitment, outcome assessment, and data analysis remained blinded to group assignments throughout the study. Group codes were disclosed only after the completion of the final participant’s assessments and statistical analysis. This method ensured allocation concealment and minimized the risk of selection and detection bias.

### Interventions

The patients’ knees were divided into two groups. Both groups received conventional physiotherapy, which consists of hot packs (HP, hydrocolloid-filled pads at 38–40 °C), conventional TENS (50–100 msec, 60–80 Hz), and exercise. HP and TENS were applied simultaneously for 20 min, while patients were positioned supine with their knees flexed at 30 degrees.

For Group 1, active HILT was applied using the “gonarthrosis protocol” of the BTL-6000 High-Intensity Laser (10 W, 1064 nm, March 2021, Hertfordshire, England). The therapy was delivered in scanning mode over the medial aspect of the knee joint. The protocol consisted of four sequential phases: pulsed (analgesic) mode for 30 s, short pulse for 1 min and 3 s, triangular monophasic pulse for 2 min and 6 s, and continuous (biostimulant) mode for 2 min and 26 s. Per session, the laser delivered a maximum power of 10.0 W, a mean power of 5.7 W, a dose of 99 J/cm², and a total energy flux of 2079 J over 6 min and 5 s, applied on a 21 cm² treatment area (Table 1).

**Table 1:** Gonarthrosis mode of HILT^*^ procedure per session

Modes (in sequence)	Intensity	Power	Duration
1. Pulsed analgesic	45 J / cm^2^	10.0 W	30 s
2. Single pulse	3 J / cm^2^	10.0 W	1 min 3 s
3. Triangular monophasic (TMP)	30 J / cm^2^	10.0 W	2 min 6 s
4. Continuous	62 J / cm^2^	8.9 W	2 min 6 s
**TOTAL**	**99 J / cm** ^**2**^ **(Total 2079 J)**	**Mean: 9,7 W** **Max: 10 W**	**6 min 5 s**

^*^HILT, High-intensity laser therapy

W, Watt

For Group 2, as control, placebo HILT was administered via the “demo protocol” where the probe light was on but no laser beam was emitted. The duration of application was the same as for Group 1, and the device was positioned to prevent the patient from viewing the screen.

Patients in both the active and control groups were asked to wear protective eyewear during HILT sessions. All interventions in both groups were conducted for 5 consecutive days over 2 weeks, 10 sessions in total.

All patients were instructed to perform joint range of motion exercises, hamstring and quadriceps stretching, and quadriceps isometric and isotonic strengthening. Each exercise was recommended to be performed 10 times per set, twice daily.

Throughout the study period, the authors did not prescribe any type of analgesic or anti-inflammatory medicine for knee pain in order not to affect the results of the study.

### Radiographic evaluation

Radiographic evaluation of the knees was performed using weight-bearing anteroposterior and lateral X-ray images of the knee joints. The severity of KOA was assessed based on joint space narrowing (JSN), which was described using the Kellgren-Lawrence system: grade 0 = no evidence of osteophytes or JSN; grade 1 = suspicious, but no definite osteophytes or JSN; grade 2 = definite osteophytes with or without possible JSN, or definite mild (less than 50%) JSN with or without osteophytes; grade 3 = moderate (at least 50%) JSN with cysts or sclerosis and usually osteophytes; and grade 4 = severe JSN with definite osteophytes, cysts, sclerosis, or deformity [24].

### Outcome measures

Pain, stiffness, maximum walking distance, and daily functional status were assessed with the Visual Analog Scale (VAS), Western Ontario & McMaster Universities Osteoarthritis Questionnaire (WOMAC) and Lequesne Algofunctional Index (LAI).

VAS is based on patients marking their pain level on a 100 mm-long straight line (0 = no pain; 100 = maximal pain) [25]. WOMAC is a self-administered osteoarthritis index comprising three subscales (pain, stiffness, and physical function subscales) and 24 questions rated on a Likert scale [26]. LAI is an interviewer-administered questionnaire, which consists of three scales (pain or discomfort, maximum walking distance, and daily living activities) comprising 10 items [27]. Both WOMAC and LAI are reliable for the assessment of KOA pain and functional status and were validated in the Turkish population [28]. Higher scores on all three questionnaires indicate poorer functional capacity and greater pain and stiffness.

Assessments were performed just before treatment, immediately at the end of the 10th session, and 12 weeks after the last therapy session. For bilateral KOA, all interventions and assessments were conducted separately for each knee at different times.

### Statistical analyses

Statistical analyses were performed the Statistical Package of Social Science (SPSS) version 25.0 (IBM Corp., Armonk, NY, USA) and jamovi (Version 2.4). The conformity of the variables to normal distribution was analyzed by the Shapiro-Wilk test. Mean, standard deviation, median, minimum and maximum values were used for descriptive analyses. Intraobserver and interobserver agreement for Kellgren-Lawrence staging was measured using Cohen’s kappa goodness-of-fit statistic. The Mann Whitney U Test was used to evaluate the variables that did not have normal distribution between placebo and HILT groups. Frequency and percentage values for the variables were used when presenting categorical variables. The relationships between categorical variables were analyzed by Chi-Square and Fisher-Freeman-Halton Exact Test. The mixed effects model was used to investigate significant differences between placebo and HILT groups in terms of repeated measures. Differences between groups were determined by Dunn’s Bonferroni test. P-values below 0.05 were accepted as statistically significant results.

## Results

**Fig. 1:**
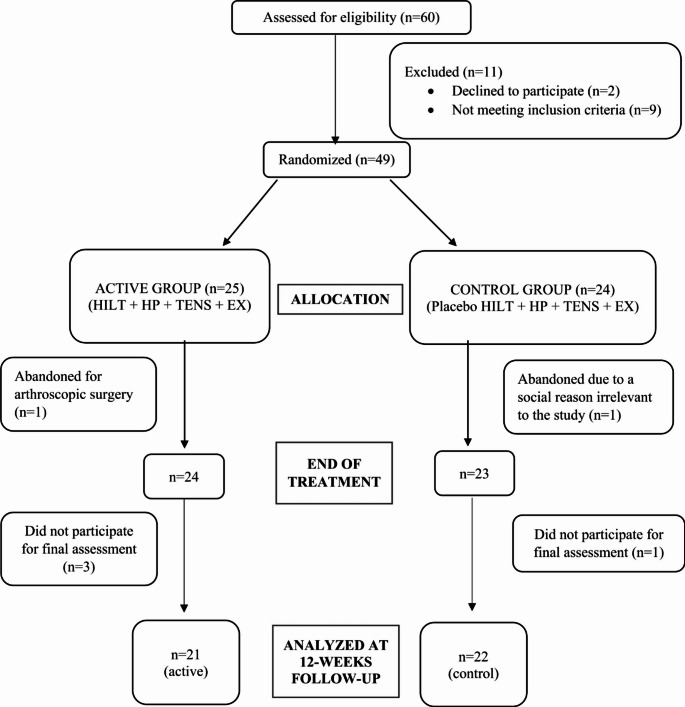
Flow diagram for the study

Initially, 60 knees from 48 patients were suitable for the study. After exclusions (9 not meeting inclusion criteria, 2 did not want to participate, 2 left during sessions, and 4 did not attend the 3-month follow-up), the final assessment was performed on 43 knees from 31 patients. Twenty-one knees were assessed in Group 1 (HILT + HP + TENS + EX), while 22 were assessed in Group 2 (placebo HILT + HP + TENS + EX) (Fig. 1).

Demographic features of the participants were similar between the groups (*p* > 0.05). The mean age was 54.6 ± 6.22 years (range 41–64), the mean body mass index (BMI) was 32.5 ± 5.13, and the female ratio was 79.1%. 53.49% of the knees were Kellgren Lawrence (KL) grade 2, and rest were KL grade 3 KOA. Also, there were no differences between the groups in terms of baseline VAS, WOMAC total, WOMAC sub-scores, LAI total, and LAI sub-scores (*p* = 1.000) (Table 2).

**Table 2:** Demographic and baseline clinical features of the participants

		Group 1 (*n*:21) HILT + HP + TENS + Ex	Group 2 (*n*:22) Placebo HILT + HP + TENS + Ex	*p*-value
		Mean ± SD	Mean ± SD	
**Age (years)**		55.1 ± 5.9	54.1 ± 6.8	0.534
**BMI (kg/m** ^**2**^ **)**		31.82 ± 4.41	33.10 ± 5.76	0.560
**Symptom onset (months)**		44.44 ± 22.90	68.78 ± 70.38	0.628
**Gender, n(%)**	Female	14 (66.67)	20 (90.91)	0.056
	Male	7 (33.33)	2 (9.09)	
**Side, n(%)**	Left	10 (47.62)	13 (59.09)	0.451
	Right	11 (52.38)	9 (40.91)	
**KL Grade, n(%)**	2	12 (57.14)	13 (59.09)	0.897
	3	9 (42.86)	9 (40.91)	
**VAS**		63.1 ± 19.78	60 ± 24.49	1.000
**WOMAC**				
Pain		8.76 ± 3.90	8.23 ± 3.49	1.000
Stiffness		1.95 ± 1.86	2.23 ± 2.02	1.000
Function		27.29 ± 11.62	31.27 ± 12.01	1.000
Total		38 ± 16.36	41.73 ± 16.07	1.000
**LEQUESNE**				
Pain		4.38 ± 1.5	4.59 ± 1.84	1.000
Walk distance		1.81 ± 1.40	2.00 ± 1.54	1.000
Daily function		3.71 ± 0.90	4.23 ± 1.45	1.000
Total		9.9 ± 3.11	10.82 ± 4.03	1.000

Chi-Square test, Mann Whitney U test

HILT, High intensity laser therapy;

HP, Hotpack

TENS, Transcutaneous electrical nerve stimulation; Ex, exercise

KL, Kellgren lawrence

VAS, Visual analog scale

WOMAC, Western ontario & McMaster universities osteoarthritis questionnaire

KL grading was performed by two different authors at a one-week interval, and the ICC correlation coefficient was high (0.81 < K < 1.00).

Considering the VAS, WOMAC total, WOMAC pain, WOMAC stiffness, WOMAC physical function, LAI total, LAI pain (*p* < 0.001, respectively), and LAI activities of daily living (*p* < 0.012) sub-scores of both groups, there was a statistically significant decrease observed at the end of the 10th session and after 12 weeks compared to pre-treatment values. Exceptionally, the LAI-walking distance sub-score did not vary statistically significantly (*p* = 0.415) (Table 3).

**Table 3:** Comparison of time-dependent measurements in groups

		Groups		*p* values		
		HILT (mean ± sd)	Placebo (mean ± sd)	*p* (time)	*p* (group)	*p* (time*group)
VAS	Pre-treatment Post-treatment 12 weeks follow-up	63,1 ± 19,78^a^ 37,62 ± 25,28^b^ 35,95 ± 34,34^b^	60 ± 24,49^a^ 28,86 ± 22,73^b^ 33,64 ± 28,04^b^	**< 0.001**	0.402	0.758
WOMAC Pain	Pre-treatment Post-treatment 12 weeks follow-up	8,76 ± 3,90^a^ 5,62 ± 3,77^b^ 4,9 ± 4,22^b^	8,23 ± 3,49^a^ 5,05 ± 3,50^b^ 4,55 ± 4,01^b^	**< 0.001**	0.594	0.981
WOMAC Stiffness	Pre-treatment Post-treatment 12 weeks follow-up	1,95 ± 1,86 1,14 ± 1,65 0,95 ± 1,50	2,23 ± 2,02^a^ 1,45 ± 1,41^a, b^ 0,55 ± 1,34^b^	**< 0.001**	0.863	0.412
WOMAC Daily Functions	Pre-treatment Post-treatment 12 weeks follow-up	27,29 ± 11,62^a^ 19,19 ± 14,26^b^ 18,86 ± 14,90^b^	31,27 ± 12,01^a^ 20,5 ± 12,91^b^ 19,77 ± 12,48^b^	**< 0.001**	0.520	0.688
WOMAC Total	Pre-treatment Post-treatment 12 weeks follow-up	38 ± 16,36^a^ 25,95 ± 18,32^b^ 24,71 ± 19,82^b^	41,73 ± 16,07^a^ 27 ± 16,43^a^ 24,86 ± 16,94^b^	**< 0.001**	0.700	0.769
Lequesne Pain	Pre-treatment Post-treatment 12 weeks follow-up	4,38 ± 1,50^a^ 3,14 ± 2,06^a, b^ 2,67 ± 2,39^b^	4,59 ± 1,84^a^ 2,86 ± 1,91^b^ 2,45 ± 1,84^b^	**< 0.001**	0.843	0.664
Lequesne Walk Distance	Pre-treatment Post-treatment 12 weeks follow-up	1,81 ± 1,40 1,57 ± 1,25 1,71 ± 1,38	2 ± 1,54 1,82 ± 1,01 1,64 ± 1,05	0.415	0.704	0.663
Lequesne Daily Functions	Pre-treatment Post-treatment 12 weeks follow-up	3,71±,90 3,29 ± 1,76 2,71 ± 1,74	4,23 ± 1,45 3,55 ± 1,74 3,82 ± 1,89	**0.012**	0.117	0.209
Lequesne Total	Pre-treatment Post-treatment 12 weeks follow-up	9,9 ± 3,11^a^ 8 ± 4,40^a, b^ 7,1 ± 4,96^b^	10,82 ± 4,03^a^ 8,23 ± 3,49^b^ 7,91 ± 4,05^b^	**< 0.001**	0.522	0.810

Mixed effect model, for each variable, the differences between the measurements found to be significant as a result of Dunn’s Bonferroni test for pretreatment, posttreatment and 12 weeks in the HILT and placebo groups were indicated by letters such as a, b. *Different letters indicate statistical difference within the group (p < 0.05), while similar letters indicate statistical similarity*

HILT, High intensity laser therapy; HP, hotpack

VAS, Visual analog scale

WOMAC, Western ontario & McMaster universities osteoarthritis questionnaire

When comparing post-treatment values between the end of the 10th session and the 12-week follow-up, no significant changes were observed in VAS, WOMAC total score, WOMAC sub-scores, LAI total score, and LAI sub-scores for both groups (*p* > 0.05) (Table 3).

When the time-dependent changes between Group 1 and Group 2 for VAS, WOMAC total and subscores, and LAI total and sub-scores were compared, no statistically significant differences were observed either at the end of the 10 sessions or at the end of 12 weeks (*p* > 0.05) (Table 3).

No treatment-related adverse events were observed throughout the entire study period.

## Discussion

According to this study, 10 consecutive sessions of hot pack and TENS, combined with home-based knee exercises, significantly improved pain, stiffness, and daily living activities in patients with intermediate stage KOA over 3 months. An additional 10 sessions of HILT did not provide any statistically significant benefits on pain, stiffness, and function.

The effectiveness of HILT for pain and disability is influenced by region of the body and variables in treatment protocols such as periods of application, number of sessions, optimal dosages and usage options like skin color and subcutaneous thickness. In the current literature, there is a lack of consensus regarding these variables. Some findings favor HILT on the knee and shoulder [29], while others favor HILT targeting the neck and back regions [14]. Among KOA patients, the numbers and periods of HILT sessions employed varied in different studies, with some conducting consecutive applications over ten [12, 21] or seven days [19], while some administered 9 sessions every other day [23] or 12 sessions twice per week [30]. Both this study and the study by Ekici et al. found no significant difference in VAS and WOMAC scores with HILT treatment, despite discrepancies in session numbers [23]. Some investigators reported significant improvements for pain, stiffness or function, favoring HILT treatment in their studies [12, 16, 19, 21, 30]. Therefore, attributing the effectiveness of HILT solely to the numbers and/or periods of sessions may not provide sufficient evidence when evaluating its efficacy for KOA treatment. Caution may be necessary when discussing the results due to potential risk of bias in studies [10, 14, 29].

In the double-blind controlled trial by Ekici et al., patients with KOA were divided into two groups with 30 participants each. The control groups in both studies are similar, but they performed exercises under supervision. The interventions in their study were administered three times a week for three weeks, totaling 15 sessions, whereas the current study involved 10 sessions on consecutive days. As in our study, they did not perform any deep diathermy, such as ultrasound (US) or microwave. Similar to our findings, there were no statistically significant differences between the active HILT group and the control group in terms of pain and functional scores, or additionally in terms of isokinetic muscle power [23]. In the study conducted by Nazari et al. with 93 patients with KOA, one group received only HILT while another group underwent conventional physiotherapy (CPT), consisting of US and TENS, for 12 sessions every other day. These were compared with a control group that was given only exercise. At the end of the early treatment period, both HILT and CPT groups showed positive improvements in pain, range of motion, walking, and daily functions compared to the control group. However, there was no significant difference between the treatment groups. In long-term follow-up, the HILT group demonstrated significantly better results, particularly for the WOMAC stiffness subscale, compared to the CPT group. The intervention consisted of 12 sessions every other day. The use of deep diathermy, the absence of obese patients, and the different number and frequency of sessions may explain the differences in results compared to our study [16].

The mean age of the patients in this study was 54.6 years, which is slightly lower than in other studies. The exclusion of the population aged 65 and over explains this difference [16, 18, 19, 21, 23]. This study’s gender distribution favored the female gender, as seen in almost all studies related to KOA [15, 18, 19, 21, 23]. Both in this study and others which declared the mean BMI of the study groups, the majority of patients fall into the “overweight” or “obese” category [16, 21, 23]. This finding is consistent with the higher incidence of KOA among individuals who are overweight [2].

The relatively small sample size of the groups constitutes the primary limitation of the current study. We provided exercise training to the patients, but we did not supervise their exercise sessions. This may also be considered another limitation. However, this approach is more realistic, as in their actual lives, patients mostly follow their exercise routines without supervision or any feedback. Another issue to note is that if the patients used any medications for other reasons, such as headaches or toothaches, it could have impacted the results. In addition, the effectiveness of the blinding procedure was not formally evaluated using a specific questionnaire. Nevertheless, none of the participants reported recognizing their group allocation, and the routine application of a 20-minute hot pack prior to the intervention may have helped mask any thermal sensation potentially caused by the active laser treatment.

As a conclusion, adding daily high-intensity laser therapy (HILT) to a conventional physiotherapy and exercise program did not result in statistically significant improvements in pain, stiffness, or functionality in patients under the age of 65 with stage 2 or 3 knee osteoarthritis. More specific trials are needed to determine the optimal dose, frequency, and number of HILT treatments for KOA patients.

## Data Availability

No datasets were generated or analysed during the current study.

## Declarations

### Previous presentations

This paper has not been a part of any presentation to a professional association.

## References

[1] Cross M, Smith E, Hoy D et al (2014) The global burden of hip and knee osteoarthritis: estimates from the global burden of disease 2010 study. Ann Rheum Dis 73(7):1323–1330 24553908 10.1136/annrheumdis-2013-204763

[2] Sabha M, Hochberg MC (2022) Non-surgical management of hip and knee osteoarthritis; comparison of ACR/AF and OARSI 2019 and va/dod 2020 guidelines. Osteoarthr Cartil Open 4(1):100232. 10.1016/j.ocarto.2021.100232. 36474466 10.1016/j.ocarto.2021.100232 PMC9718349

[3] Bannuru RR, Osani MC, Vaysbrot EE et al (2019) OARSI guidelines for the non-surgical management of knee, hip, and polyarticular osteoarthritis. Osteoarthritis Cartilage 27(11):1578–1589. 10.1016/j.joca.2019.06.011. 31278997 10.1016/j.joca.2019.06.011

[4] Kolasinski SL, Neogi T, Hochberg MC et al (2020) 2019 American college of rheumatology/arthritis foundation guideline for the management of osteoarthritis of the hand, hip, and knee. Arthritis Rheumatol 72(2):220–233 31908163 10.1002/art.41142 PMC10518852

[5] Hardie EM, Carlson CS, Richardson DC (1989) Effect of nd:yag laser energy on articular cartilage healing in the dog. Lasers Surg Med 9(6):595–601. 10.1002/lsm.1900090610. 2601554 10.1002/lsm.1900090610

[6] Spivak JM, Grande DA, Ben-Yishay A, Menche DS, Pitman MI (1992) The effect of low-level nd:yag laser energy on adult articular cartilage in vitro. Arthroscopy: J Arthroscopic Relat Surg 8(1):36–43. 10.1016/0749-8063(92)90133-V. 10.1016/0749-8063(92)90133-v 1550649

[7] Herman JH, Khosla RC (1989) Nd: YAG laser modulation of synovial tissue metabolism. Clin Exp Rheumatol 7(5):505–512 2591125

[8] Jankaew A, You YL, Yang TH, Chang YW, Lin CF (2023) The effects of low-level laser therapy on muscle strength and functional outcomes in individuals with knee osteoarthritis: a double-blinded randomized controlled trial. Sci Rep 13(1):165 36599881 10.1038/s41598-022-26553-9 PMC9812996

[9] Malik S, Sharma S, Dutta N, Khurana D, Sharma RK, Sharma S (2023) Effect of low-level laser therapy plus exercise therapy on pain, range of motion, muscle strength, and function in knee osteoarthritis–a systematic review and meta-analysis. Somatosens Mot Res 40(1):8–24 36576096 10.1080/08990220.2022.2157387

[10] Cai P, Wei X, Wang W, Cai C, Li H (2023) High-intensity laser therapy on pain relief in symptomatic knee osteoarthritis: a systematic review and meta-analysis. J Back Musculoskelet Rehabil 36(5):1011–1021. 10.3233/BMR-220228 37458008

[11] Tim CR, Martignago CCS, Assis L et al (2022) Effects of photobiomodulation therapy in chondrocyte response by in vitro experiments and experimental model of osteoarthritis in the knee of rats. Lasers Med Sci Published Online 1–10 10.1007/s10103-021-03417-8 34554354

[12] Štiglić-Rogoznica N, Stamenković D, Frlan-Vrgoč L, Avancini-Dobrović V, Schnurrer-Luke Vrbanić T (2011) Analgesic effect of high intensity laser therapy in knee osteoarthritis. Coll Antropol 35(2):183–185 22220431

[13] Wyszyńska J, Bal-Bocheńska M (2018) Efficacy of high-intensity laser therapy in treating knee osteoarthritis: a first systematic review. Photomed Laser Surg 36(7):343–353. 10.1089/pho.2017.4425. 29688827 10.1089/pho.2017.4425

[14] Song HJ, Seo HJ, Lee Y, Kim SK (2018) Effectiveness of high-intensity laser therapy in the treatment of musculoskeletal disorders: a systematic review and meta-analysis of randomized controlled trials. Medicine 97(51). https://journals.lww.com/md-journal/fulltext/2018/12210/effectiveness_of_high_intensity_laser_therapy_in.4.aspx. 10.1097/MD.0000000000013126 PMC6319951 30572425

[15] Kheshie AR, Alayat MSM, Ali MME (2014) High-intensity versus low-level laser therapy in the treatment of patients with knee osteoarthritis: a randomized controlled trial. Lasers Med Sci 29:1371–1376 24487957 10.1007/s10103-014-1529-0

[16] Nazari A, Moezy A, Nejati P, Mazaherinezhad A (2019) Efficacy of high-intensity laser therapy in comparison with conventional physiotherapy and exercise therapy on pain and function of patients with knee osteoarthritis: a randomized controlled trial with 12-week follow up. Lasers Med Sci 34(3):505–516. 10.1007/s10103-018-2624-4. 30178432 10.1007/s10103-018-2624-4

[17] Kaub L, Schmitz C (2023) Comparison of the penetration depth of 905 Nm and 1064 Nm laser light in surface layers of biological tissue ex vivo. Biomedicines 11(5):1355 37239026 10.3390/biomedicines11051355 PMC10216207

[18] Siriratna P, Ratanasutiranont C, Manissorn T, Santiniyom N, Chira-Adisai W (2022) Short-term efficacy of high-intensity laser therapy in alleviating pain in patients with knee osteoarthritis: a single-blind randomised controlled trial. Pain Res Manag.;2022 10.1155/2022/1319165 PMC9616657 36313402

[19] Angelova A, Ilieva EM (2016) Effectiveness of high intensity laser therapy for reduction of pain in knee osteoarthritis. Pain Res Manag.;2016 10.1155/2016/9163618 PMC5206453 28096711

[20] Kim GJ, Choi J, Lee S, Jeon C, Lee K (2016) The effects of high intensity laser therapy on pain and function in patients with knee osteoarthritis. J Phys Ther Sci 28(11):3197–3199. 10.1589/jpts.28.3197. 27942148 10.1589/jpts.28.3197 PMC5140828

[21] Akaltun MS, Altindag O, Turan N, Gursoy S, Gur A (2021) Efficacy of high intensity laser therapy in knee osteoarthritis: a double-blind controlled randomized study. Clin Rheumatol 40(5):1989–1995. 10.1007/s10067-020-05469-7. 33074393 10.1007/s10067-020-05469-7

[22] Samaan SSRR, Sedhom MG, Grace MO (2022) A randomized comparative study between high-intensity laser vs low-intensity pulsed ultrasound both combined with exercises for the treatment of knee osteoarthritis. Int J Rheum Dis 25(8):877–886. 10.1111/1756-185X.14361. 35678062 10.1111/1756-185X.14361

[23] Ekici B, Ordahan B (2023) Evaluation of the effect of high-intensity laser therapy (HILT) on function, muscle strength, range of motion, pain level, and femoral cartilage thickness in knee osteoarthritis: randomized controlled study. Lasers Med Sci 38(1):218 37743421 10.1007/s10103-023-03887-y

[24] Analan PD, Ozdemir H (2020) The effect of patellar height by using insall Salvati index on pain, function, muscle strength and postural stability in patients with primary knee osteoarthritis. Curr Med Imaging Former Curr Med Imaging Reviews 17(4):532–538. 10.2174/1573405616999200817172649. 10.2174/1573405616999200817172649 32811402

[25] Alghadir AH, Anwer S, Iqbal A, Iqbal ZA (2018) Test–retest reliability, validity, and minimum detectable change of visual analog, numerical rating, and verbal rating scales for measurement of osteoarthritic knee pain. J Pain Res Published Online 851–856 10.2147/JPR.S158847 PMC5927184 29731662

[26] Woolacott NF, Corbett MS, Rice SJC (2012) The use and reporting of WOMAC in the assessment of the benefit of physical therapies for the pain of osteoarthritis of the knee: findings from a systematic review of clinical trials. Rheumatology 51(8):1440–1446 22467082 10.1093/rheumatology/kes043

[27] Lequesne M (1991) Indices of severity and disease activity for osteoarthritis. Semin Arthritis Rheum 20(6):48–54. 10.1016/0049-0172(91)90027-W. 1866630 10.1016/0049-0172(91)90027-w

[28] Basaran S, Guzel R, Seydaoglu G, Guler-Uysal F (2010) Validity, reliability, and comparison of the WOMAC osteoarthritis index and Lequesne algofunctional index in Turkish patients with hip or knee osteoarthritis. Clin Rheumatol 29:749–756 20169459 10.1007/s10067-010-1398-2

[29] Arroyo-Fernández R, Aceituno-Gómez J, Serrano-Muñoz D, Avendaño-Coy J (2023) High-intensity laser therapy for musculoskeletal disorders: a systematic review and meta-analysis of randomized clinical trials. J Clin Med 12(4):1479 36836014 10.3390/jcm12041479 PMC9963402

[30] Alayat MSM, Aly THA, Elsayed AEM, Fadil ASM (2017) Efficacy of pulsed nd: YAG laser in the treatment of patients with knee osteoarthritis: a randomized controlled trial. Lasers Med Sci 32:503–511 28078503 10.1007/s10103-017-2141-x

